# What Do Lithuanian Hunters Think of African Swine Fever and Its Control—Perceptions

**DOI:** 10.3390/ani11020525

**Published:** 2021-02-18

**Authors:** Evelina Stončiūtė, Katja Schulz, Alvydas Malakauskas, Franz J. Conraths, Marius Masiulis, Carola Sauter-Louis

**Affiliations:** 1 Department of Veterinary Pathobiology, Veterinary Academy, Lithuanian University of Health Sciences, Tilzes 18, LT-47181 Kaunas, Lithuania; Alvydas.Malakauskas@lsmuni.lt; 2 Friedrich-Loeffler-Institut, Federal Research Institute for Animal Health, Institute of Epidemiology, Südufer 10, 17493 Greifswald, Germany; katja.schulz@fli.de (K.S.); Franz.Conraths@fli.de (F.J.C.); Carola.Sauter-Louis@fli.de (C.S.-L.); 3 Emergency Response Department, State Food and Veterinary Service, Siesiku 19, LT-07170 Vilnius, Lithuania; marius.masiulis@vmvt.lt; 4 Dr. L Kriauceliunas Small Animal Clinic, Veterinary Academy, Lithuanian University of Health Sciences, Tilzes Str.18, LT-47181 Kaunas, Lithuania

**Keywords:** African swine fever, wild boar, acceptability, surveillance

## Abstract

**Simple Summary:**

Effectiveness and successful implementation of control measures greatly depend on hunters’ willingness and motivation to carry out these measures. Therefore, assessing their opinions regarding the current system is paramount in order to achieve the best possible results. The present study provides insights of hunters’ attitudes and perceptions about measures to control African swine fever (ASF) in wild boar in Lithuania. This study highlights several measures that are not supported by hunters (i.e., restriction of hunting, selective female hunting, ban of supplementary feeding, involvement of additional forces in ASF control) and possible motivational options (i.e., reduction of work, financial incentives and improved feedback and relationships with government officials). Considering these findings when planning and altering control measures for ASF could improve their successful implementation in the field.

**Abstract:**

After the introduction of African swine fever (ASF) into Lithuania in 2014, continuous spread of the disease resulted in infection of the wild boar populations in most parts of Lithuania. The virus has been moving closer to other Western European countries where pig density is high. An efficient surveillance system detecting ASF cases early in domestic and wild animals is necessary to manage this disease. To make surveillance appropriate and effective, it is critical to understand how key players perceive the implemented control measures. This study investigated the attitudes and beliefs of hunters in Lithuania regarding currently implemented or proposed measures for the control of ASF in the wild boar population. Study data were collected through questionnaires distributed via the internet and by hunting associations in Lithuania. In total, 621 fully completed questionnaires were received and analyzed. All measures interfering with extensive hunting, like ban of driven or individual hunting or ban of supplementary feeding were considered as unacceptable and as ineffective measures to control ASF in wild boar. However, selective hunting of female wild boar was generally considered as an unethical act and therefore rejected. Some measures that seem to have been successful in other countries, like involvement of additional forces, were rejected by Lithuanian hunters, thus implementation of these measures could be difficult. The study highlighted that there is a need for improving important relationships with other stakeholders, since many hunters expressed a lack of trust in governmental institutions and regarded cooperation with them as insufficient. Hunters emphasized that their motivation to support passive surveillance measures could be improved with financial compensation and reduction of workload. The present study provides insights into hunters’ perceptions, which may be used as a foundation for additional discussions with these important stakeholders and for adapting measures to improve their acceptance if appropriate.

## 1. Introduction

African swine fever (ASF) is a virulent disease that poses a major threat to animal health and trade in many European Union countries and is one of the most important viral diseases for the pig industry [1,2]. The disease is induced by a large complex, highly virulent DNA virus that can affect domestic pigs and wild boars [3,4]. In infected animals, clinical signs of ASF vary considerably from peracute death to acute hemorrhagic disease with severe pathomorphological changes. Currently, no effective vaccine is available [4,5,6,7]. Due to high case/fatality ratio and the ability of ASF to rapidly spread within piggeries, outbreaks result in significant costs, e.g., due to the need of culling all pigs in affected farms [8]. The presence of ASF virus (ASFV) in domestic pigs and wild boars in a country also leads to trade restrictions on pork products at international level, which may cause substantial losses for the pig sector in the national economy [9]. ASFV genotype II, affecting Eastern European countries and the Baltic States, was first introduced into Georgia in 2007 and spread through the Caucasus and the Russian Federation [5,10]. In 2014, first cases of the virus were confirmed in the EU, i.e., in Lithuania, Latvia, Estonia and Poland [11]. Since 2018, ASFV has been present in wild boar populations in most parts of Lithuania [12]. Wild boar population plays an important role in ASFV spread and maintaining the virus in the environment [13,14]. The presence of ASF in wild boar populations is one of the main risk factors for domestic pig farms to become infected with the virus [6,15,16,17]. While soft ticks of the genus *Ornithodoros* have shown to be competent vectors for ASFV and can maintain the virus in the environment in African countries and played some role in ASF transmission on the Iberian Peninsula in the past, their geographical distribution in Eurasia is mainly limited to the Mediterranean Basin, to Transcaucasian countries and some parts of Russian Federation [7]. *Ornithodoros* ticks do not occur in the Baltic states and they play no role in ASFV transmission in this region. In several countries, the first ASF cases were detected in wild boar [5]. Thus, disease surveillance in wild boar constitutes an important strategy to fight ASF effectively [13]. To ensure early detection of an ASFV introduction, passive surveillance (i.e., the sampling of wild boar found dead or shot sick), is of utmost importance [11,15,18,19,20]. Since hunters are the crucial contributors for passive surveillance in wild boar, knowledge about their attitudes and willingness to participate in the implemented and proposed measures is essential [21]. Therefore, their views are needed to revise and to improve current surveillance systems, and to increase the chances of their successful implementation. To give hunters the chance to express their opinion freely, the survey was conducted anonymously. The present study aimed to assess the perceptions, concerns of hunters and their willingness to support ASF control measures that are currently implemented in Lithuania.

## 2. Materials and Methods 

### 2.1. Design of Questionnaire

The questionnaire was designed in Lithuanian language and administered to hunters in a web-based survey conducted from 8 July to 2 August 2020. During a pilot study, a draft questionnaire had been circulated among a group of six hunters to evaluate its length and the clarity of the given questions. Based on the feedback received during this pilot study, the questionnaire was modified and improved. Questions in the survey were mainly single- or multiple-choice questions and most of them had an open-ended response choice that allowed hunters to include their personal observations. In three questions (1, 7, and 10) a five-point Likert scale was used [22]. The final questionnaire is presented in Supplementary Material, S1.

The questionnaire comprised four parts.

1. General information: Gender, age group, county the respondents live in, hunting frequency (per month) and hunting experience (in years).
2. Knowledge about ASF: The perceived knowledge of the respondents regarding ASF was assessed.
3. Control of ASF: Hunters had to evaluate the individual control measures against ASF by assessing their feasibility and perceived effectiveness.
4. Enhanced passive surveillance: Respondents stated their perceptions of the effectiveness of passive surveillance within ASF control and their willingness to support carcass search.

### 2.2. Distribution of Questionnaire

The study was implemented as anonymous web-based survey. An invitation letter with the link to the questionnaire on a SurveyMonkey platform (www.surveymonkey.com, accessed date: 8 July 2020) was distributed via email to the five main hunting associations in Lithuania and by posting the invitation on the most frequently visited website by hunters in Lithuania (www.miske.lt, accessed date: 13 July 2020). Prior to the distribution, an official representative of the hunters’ associations and website administrator were contacted by phone in order to explain the purpose of the study, the use of the results, and the anonymous response nature. Hunters associations distributed the invitation to their regional offices, which were asked to forward it to their members. The email invitations to the associations also contained the questionnaire in a Word file in case this would have been the preferred way to respond for some hunters. In the questionnaire, hunters were first informed about the purpose of the study and asked for their consent. Browser cookies ensured unique responses from individual hunters answering the web-based survey. By participating in the survey or returning the completed questionnaire, the participants agreed to an anonymous use of the information they provided for research and publication purposes. 

### 2.3. Questionnaire Analysis

Questionnaires received through the online survey or as Word files were collected and analyzed using the open software R (Version 1.2.1335, http://www.r-project.org (accessed on 17 February 2021)). Descriptive statistics such as frequencies and percentages were calculated using the basic functions in R. Wilson 95% confidence intervals (95% CI) were calculated for these percentages using the “binom” package [23]. The following potential associations were analyzed: Age versus perceived knowledge about ASF, versus hunting experience and hunting frequency; hunters’ knowledge about ASF versus hunting frequency and versus experience; perceptions, whether ASF control and elimination was regarded as feasible versus respondents’ county and hunting frequency. Participation in selective wild boar female hunting versus demographic data like age, county and hunting experience were analyzed using frequency distributions. Univariable associations were examined using chi-squared tests or Fisher’s exact tests, if at least one expected count was less than 5. *p*-values of less than 0.05 were considered statistically significant.

## 3. Results

### 3.1. General Information 

A total of 786 questionnaires were filled in by hunters online, 617 (78.50%) of them were fully completed and were included in further analysis. Four additional completed questionnaires were received in a Word file from members of the Lithuanian hunting associations, resulting in 621 questionnaires suitable for analysis. Descriptive statistics and frequency distributions are shown in Appendix A, Table A1. The majority of respondents (97.58%, CI: 96–99%) were male hunters. Most participants (48.47%, CI: 45–52%) were in the age group of 41–60 years. Hunters originated from all counties of Lithuania, but most of them were from the larger Lithuanian counties (i.e., Vilnius, Kaunas and Šiauliai). The respondents’ hunting frequency varied between 1–5 times per month (47.02%, CI: 43–51%) and 6–10 times per month (33.17%, CI: 30–37%). The majority of the respondents (69.89%, CI: 66–73%) had more than 10 years of hunting experience (Appendix A, Table A1).

### 3.2. Knowledge about ASF

#### 3.2.1. Assessment of Hunters’ Perceived Knowledge about ASF

The majority of respondents (41.55%, CI: 38–45%) strongly agreed to the statement that their knowledge about ASF is good or agreed to this statement to a high extent (41.22%, CI: 37–45%) (Table 1). 

#### 3.2.2. Listed Information Sources about ASF 

Regarding the sources for information about ASF, respondents most frequently listed the “State Food and Veterinary Service” (75.68%, CI: 72–79%), followed by “Hunting magazines” (48.47%, CI: 44–52%), “Other hunters” (45.09%, CI: 41–49%) and “Newspapers and TV” (48.79%, CI: 45–53%). In the open responses, hunters added that they mainly got additional information about ASF from the internet, social media and from their hunting associations. Some hunters listed that they were veterinarians or had other relevant educational background, others mentioned EU documents as an additional source of information about ASF. 

### 3.3. Assessment of ASF Control

#### 3.3.1. Hunters’ Participation in Actions to Control ASF 

When asked in what kind of ASF control actions the hunters had taken part, the respondents listed sampling of wild boar (79.87%, CI: 77–83%), intensified hunting (67.79%, CI: 64–71%) and performing increased biosecurity measures (66.18%, CI: 62–70%) most frequently (Table 2).

The majority of respondents stated that they participated in these actions in the years between 2018/19 (73.75%, CI: 70–77%), 2019/20 (60.23%, CI: 57–64%) and 2017/18 (53.14%, CI: 49–57%).

#### 3.3.2. Perceptions of ASF Control and the Feasibility of Elimination in Lithuania 

A total of 70% (CI: 67–74%) of the respondents believed that control and elimination of ASF can be achieved in Lithuania, and 53% (CI: 49–57%) thought that ASF will not disappear without human intervention.

#### 3.3.3. Assessment of the Effectiveness of Control Measures to Eliminate ASF

**Figure 1:**
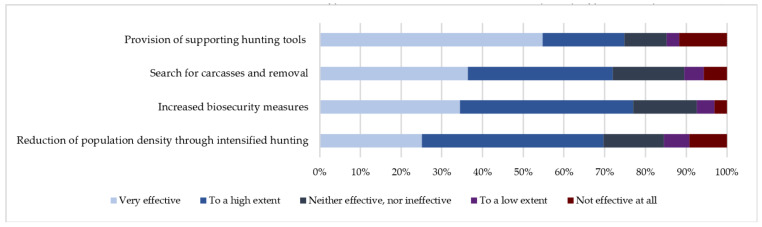
The most effective measures to eliminate ASF in wild boar population perceived by Lithuanian hunters responding to the anonymous survey.

Most respondents believed that to them the most effective measures in eliminating ASF were: Permission to use additional hunting tools (54.75% very effective, CI: 50–59%, 20.13% effective to a high extent, CI: 17–23%), search for carcasses and removal (36.39% very effective, CI: 33–40; 35.59% effective to a high extent, CI: 32–39%), increased biosecurity measures (34.46% very effective, CI: 31–38%; 42.51% effective to a high extent, CI: 39–46%) and reduction of population through intensified hunting (25.12% very effective, CI: 33–40%; 44.61% effective to a high extent, CI: 32–39%) (Figure 1).

**Figure 2:**
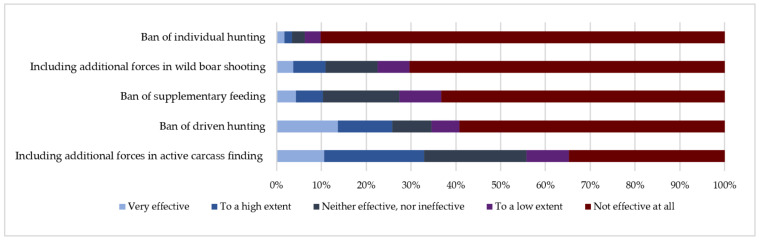
The least effective measures to eliminate ASF in wild boar population perceived by Lithuanian hunters responding to the anonymous survey.

Hunters perceived the following measures as the least effective ones: Ban of individual hunting (effective to a low extent 3.54%, CI: 2–5%; not effective at all 90.18%, CI: 88–92%), including additional forces in wild boar shooting (effective to a low extent 7.09%, CI: 5–9%; not effective at all 70.37%, CI: 67–74%), ban of supplementary feeding (effective to a low extent 9.34%, CI: 7–12%; not effective at all 63.29%, CI: 60–67%), ban of driven hunting (effective to a low extent 6.12%, CI: 4–8%; not effective at all 59.26%, CI: 56–63%) and including additional forces for active carcass search (effective to a low extent 9.5%, CI: 7–12%; not effective at all 34.78%, CI: 31–39%) (Figure 2).

#### 3.3.4. Assessment of the Least Feasible ASF Control Options in the Field

Hunters considered the inclusion of additional forces like army and police in ASF control as the least feasible option (60.71%, CI: 57–64%), followed by ban of hunting (28.02%, CI: 25–32%), selective hunting of females (24.34%, CI: 22–29%) and ban of supplementary feeding (19.81%, CI: 17–23%). The lack of appropriate skills was stated as the main reason for the perceived lack of feasibility for including additional forces in the control of ASF. Further listed reasons were other responsibilities of these forces and unnecessary extra costs. Respondents argued that a hunting ban would increase the wild boar population and worsen the ASF situation. In addition, some hunters stated that a hunting ban might greatly reduce hunters’ motivation to participate in ASF control measures overall. Ban of supplementary feeding was not considered feasible, because this was described as a necessary measure to reduce wild boar migration. Regarding the selective hunting of female wild boar, participants mainly stated that sow hunting violated ethical principles and may result in the elimination of the wild boar population. 

#### 3.3.5. Hindering Reasons to Support ASF Control and Elimination

Participants indicated that the main obstacles in supporting ASF control and elimination were time constraints (26.57%, CI: 23–30%), additional costs (23.83%, CI: 21–27%), lack of trust in the authorities (18.84%, CI: 16–22%) and internal agreements of a hunter club (18.36% CI: 15–21%). In open responses, hunters stated that hunting restrictions, limited cooperation with the governmental institutions and their presumed lack of respect for hunters were additional hindering reasons to support control and elimination of ASF. Some respondents also mentioned that they were not willing to support control measures because they thought that ASF was not a problem in their hunting grounds at the time when filling in the questionnaire.

### 3.4. Attitudes towards Passive Surveillance

#### 3.4.1. Assessment of the Effectiveness of Passive Surveillance and Hunters’ Willingness to Support It

**Figure 3:**
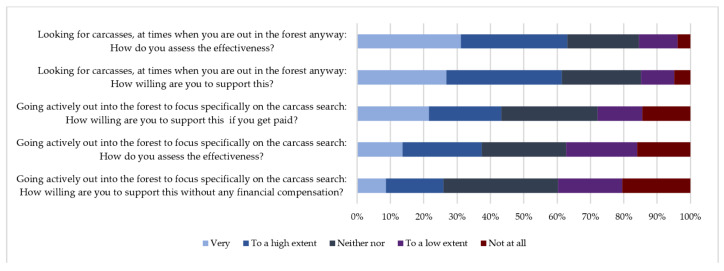
Evaluation of passive surveillance measures by Lithuanian hunters responding to the questionnaire survey.

In assessing passive surveillance measures, hunters stated that searching for wild boar carcasses while they were out in the forest anyway as a very effective measure (31.08%, CI: 28–35%), or effective to a high extent (32.05%, CI: 29- 36%). Regarding their willingness to support this measure, a similar proportion of respondents stated that they were very willing to do this (26.73%, CI: 23–30%) or willing to a high extent (34.62%, CI: 31–38%) (Figure 3).

Going into the forest specifically for wild boar carcass search was considered to be a less effective measure, compared to the previous option (13.69%, CI: 11–17% listed this to be a very effective measure and 23.67%, CI: 20–27% effective to a high extent). More hunters stated they would be willing to support this measure with compensation (21.58%, CI: 19–25% very willing, 21.74%, CI: 19–25% willing to a high extent), compared to the option without compensation (very willing−8.7%, CI: 7–11%, willing to a high extent−17.23%, CI: 14–20%). 

#### 3.4.2. Hindering Reasons to Support Passive Surveillance Measures

Respondents stated that the main hindering reason to support carcass search and removal was time constraints (50.72%, CI: 47–55%). Some respondents also listed difficulties to find dead wild boar (29.31%, CI: 26–33%) and 26.73% (CI: 23–30%) stated that they are not willing to participate because they do not believe that carcass search and removal helps in eliminating ASF. A total of 26.57% (CI: 23–30%) mentioned additional costs as a hindering reason to support carcass search and removal and 21.9% (CI: 19–25%) were not willing to support carcass search because of the requirement to bury carcasses. In the open responses, 11 hunters added that they lacked trust in government institutions, five mentioned the lack of compensation and four noted that the main hindering reason is that they have internal agreements with the authorities of the hunting club not to support these measures.

#### 3.4.3. Evaluation of Motivational Options to Support Passive Surveillance

Regarding the options, which might increase hunters’ motivation to support passive surveillance, 47.34% (CI: 43–51%) of respondents stated that they would be more motivated if there was no further work involved than reporting the location of the dead wild boar. A total of 39.13% (CI: 35–43%) of respondents thought that their motivation might increase if they got detailed feedback from the authorities about the wild boar disease status and 37.04% (CI: 33–41%) of the respondents mentioned personal financial support as a motivating factor. In the open responses, some hunters added that their motivation to support passive surveillance might increase, if they could use additional hunting tools like silencers or night vision equipment or if their activities received respect from the government.

### 3.5. Univariate Analysis Between Groups

The analysis showed that the number of respondents in the age group above 60 years stating that they have not enough knowledge about ASF, was statistically significantly lower compared to other age groups (*p* = 0.036). There was a significantly higher number of hunters with a hunting experience of more than 10 years, compared to hunters with less experience, who listed that they had participated in selective female wild boar hunting (*p* = 0.036). Other explored associations results are listed in Appendix B, Table A2.

## 4. Discussion

The objective of this study was to identify the perceptions and opinions of Lithuanian hunters regarding the measures to control and eliminate ASF, as acceptance and support of key stakeholders is paramount in ensuring the effectiveness of surveillance systems and control measures [24,25]. The questionnaire used here covered the main aspects of currently applied ASF surveillance measures and allowed the assessment of general concerns and opinions of hunters regarding ASF. The study highlighted that there is a need for improving important relationships with other stakeholders, since many hunters expressed a lack of trust in governmental institutions and regarded cooperation with them as insufficient. Hunters emphasized that their motivation to support passive surveillance measures could be improved by financial compensation and a reduction of their workload.

A total of 621 fully completed questionnaires were analyzed, representing around 2% of the Lithuanian hunting population, which consists of approximately 30,000 hunters. Due to the web-based character of the survey, it was not possible to calculate a response ratio. It is therefore not known how many hunters were reached. A considerable proportion of 72 out of the 169 respondents, who did not complete the full questionnaire, stopped when they reached the five-point Likert scale questions (question 1, 7, and 10) (See Supplementary Material, S1), what may indicate that these questions were too difficult to understand and that the respondents lost interest. Simplifying some parts of the questionnaire may thus have resulted in a higher number of completed responses. 

Our analysis showed that mostly male hunters participated in the survey. Although this may represent the current gender distribution among Lithuanian hunters, a possible gender bias in our study must be taken into account. The age group over 60 years was represented with the lowest number of participants. However, the age distribution of the respondents is in accord with the official demographic data of the age distribution of registered hunters in Lithuania, thus indicating that age was no major bias in the study. Nevertheless, we cannot exclude that some older hunters might have been more willing to participate, if they had the option to complete the questionnaire on paper rather than online.

Responses from all Lithuanian counties were recorded. If we assume that for the majority of the respondents their hunting grounds were close to their homes, we can conclude that hunters from various ASF-affected and unaffected regions participated in the survey. Therefore, different experiences regarding ASF control measures were expressed, reducing possible bias due to differences among various Lithuanian regions. Thus, we are confident, that the responders of the survey were representative of the Lithuanian hunters’ population.

Similar to findings in Estonia, where focus-group discussions took place [26], hunters in the current study believed that all measures that include hunting, like supporting additional hunting tools and intensified hunting were effective to control ASF. Although hunters considered increased biosecurity and carcass search and removal as effective measures to control this disease, many of them agreed that these measures would gain more support in the hunters‘ community, if they were reimbursed by the government for expenses occurring when performing these tasks. This result was in accord with the perceptions of hunters in Estonia [26]. As anticipated, hunters regarded measures that interfered with hunting, like ban of driven or individual hunting, as ineffective. Respondents feared that the ban of hunting would only make the current situation worse and might further reduce the hunters‘ motivation to support the ASF control measures implemented in Lithuania. Ban of supplementary feeding was also considered as a measure that interferes with an effective reduction of wild boar population, as many hunters believed that offering additional feed might keep the animals settled in one area, thus making it easier to hunt them. While hunters were willing to support all hunting-related measures, selective hunting of female wild boar was considered as unacceptable for ethical reasons and it was feared that this practice may lead to the overall extinction of the wild boar population. This result was very similar to the observations made in Estonia [26] and Latvia as well [27]. Shooting female wild boar was more acceptable for the more experienced group of hunters. This might indicate that longer involvement in hunting activities encourage hunters to participate in measures deemed unpleasant, but necessary.

While including additional forces, such as army or police, in ASF control is not a measure currently implemented in Lithuania, it was included in the questionnaire as this has been successfully practiced in other countries [28,29]. Our analysis showed that Lithuanian hunters did not support the option of including additional forces for carcass searches and shooting wild boar. Many of the respondents believed that additional forces might not have the appropriate skills required to control wild animal populations. This result is in accordance with previous findings regarding hunters‘ perceptions on involving additional staff in ASF control in Estonia [26] and in Latvia as well, where hunters also expressed that including forces like army and police would impose additional biosecurity risks [27]. In addition to time constraints and additional costs, many hunters listed a lack of trust in the authorities as main hindering reasons to support ASF control measures. Frequently, respondents mentioned that there was not enough cooperation with the government institutions regarding passive surveillance and testing procedures, also adding that they felt a lack of respect for hunters. These findings indicate that the contact between stakeholders may have to be improved, as satisfactory relationships can increase the acceptability of control measures and willingness to cooperate [25,30]. Lack of understanding of the key stakeholders’ intentions, priorities and interests may be followed by reduced support of hunters and even unwillingness to implement ASF control measures [24]. If the State Food and Veterinary Service provided continuous feedback to the hunters‘ communities about the current situation of ASF in wild boar and domestic pigs, e.g., in meetings and other interactions, this could help improving the important relationship between government institutions and hunters [31].

Passive surveillance is very important in early disease detection, since the likelihood of detecting ASFV is usually higher in found carcasses than in hunted wild boar [7,15,18,32]. It is therefore important to strengthen the success of this surveillance component by maximizing hunters’ willingness to report wild boar carcasses [31]. Hunters were thus asked to assess passive surveillance measures. Respondents were mostly willing to participate in passive surveillance measures when they were out in the forest for hunting or other outdoor activities anyway. They did not support the idea of separate outings just for carcass search and removal. Financial incentives did not change their opinion on this matter considerably. Interestingly, some hunters indicated that they did not believe that carcass search and removal can help to eliminate ASF. This stresses the significance of communication with the hunters and the importance for stakeholders to understand the reasons of currently implemented control measures. Similar to the studies conducted in Estonia [26] and Latvia [27], the most favored motivational option for hunters to support passive surveillance was a reduction of workload. Many respondents believed that their willingness to participate in these measures would increase if they had no further work than reporting where the wild boar carcass was located, instead of having to bury them in addition to reporting. Considering the importance of increasing the numbers of carcass finding for ASF surveillance and control, an acceptable solution regarding carcass removal that is acceptable to hunters, should be reached among the relevant stakeholders in Lithuania.

## 5. Conclusions

The present study provides insights into the attitudes and perceptions among Lithuanian hunters on currently implemented or proposed ASF control measures. ASF is still circulating in the wild boar populations in Lithuania, causing a constant threat to animal health and trade, thus necessitating the hunters’ support and their continuous willingness to participate in ASF surveillance and control. This study highlighted that there is a lack of knowledge in the hunters’ community regarding the reasoning behind certain ASF control measures implemented in Lithuania, which may be related to the opinion expressed by hunters that there is insufficient trust and cooperation with governmental institutions. The study also showed that in addition to financial incentives, reduction of the hunters’ workload could improve their motivation to participate in passive surveillance measures. Further analyses of hunters’ opinions regarding the implemented ASF control and elimination measures may be used to prepare more detailed recommendations for the decision makers in Lithuania in order to improve the current surveillance system and the control of ASF in wild boar.

## Figures and Tables

**Table 1:** Perception about the knowledge of ASF by Lithuanian hunters responding to the survey.

Which Response Best Describes the Extent to Which You Agree or Disagree with the Statement: My Knowledge about ASF Is Good.				
Strongly Agree *n* (%)	To a High Extent *n* (%)	Neither Agree nor Disagree *n* (%)	To a Low Extent *n* (%)	Strongly Disagree *n* (%)
258 (41.55%) (CI: 38–45%)	256 (41.22%) (CI: 37–45%)	87 (14.01%) (CI: 12–17%)	16 (2.58%) (CI: 2–4%)	4 (0.64%) (CI: 0.2–1.6%)

CI—confidence intervals.

**Table 2:** ASF control actions in which respondents to an anonymous questionnaire amongst Lithuanian hunters had taken part in.

Action	Number	% of Respondents (95% CI)
Sampling of wild boar	496	79.87% (77–83%)
Intensified hunting	421	67.79% (64–71%)
Performing increased biosecurity measures	411	66.18% (62–70%)
Voluntary active carcass search	213	34.30% (31–38%)
Disposal of carcass	199	32.05% (28–36%)
Compulsory active carcass search	185	29.79% (26–34%)
Selective hunting of female wild boar	103	16.59% (14–20%)
None	19	3.06% (2–5%)
Other	5	0.81% (0.3–2%)

## Data Availability

The data used in the analyses can be completely obtained from the author by request.

## Supplementary Materials

The following are available online https://www.mdpi.com/2076-2615/11/2/525/s1. Document S1: Questionnaire “Control of African swine fever (ASF) in wild boar”.

## Appendix A

**Table A1:** Demographic data of the Lithuanian hunters responding to the questionnaire survey.

Variable	Number	% of Respondents (95% CI)
**Gender**		
Female	15	2.47% (1–4%)
Male	606	97.58% (96–99%)
**Age-group**		
18–40 years	236	38% (34–42%)
41–60 years	301	48.47% (45–52%)
> 60 years	84	13.53% (11–16%)
**County**		
Alytaus	49	7.89% (6–10%)
Kauno	103	16.59% (14–20%)
Klaipėdos	45	7.25% (5–10%)
Marijampolės	40	6.44% (5–9%)
Panevėžio	48	7.73% (6–10%)
Šiaulių	76	12.24% (10–15%)
Tauragės	39	6.28% (5–8%)
Telšių	31	4.99% (4–7%)
Utenos	53	8.53% (7–11%)
Vilniaus	137	22.06% (19–25%)
**Hunting frequency (per month)**		
less than once	54	8.7% (7–11%)
1–5 times	292	47.02% (43–51%)
6–10 times	206	33.17% (30–37%)
more than 10 times	69	11.11% (9–14%)

## Appendix B

**Table A2:** Univariate analysis results of explored associations.

Compared Variables		Statistical Significance, *p*-Value	Direction of Association
Age	Knowledge about ASF	0.036 *	Older age groups were less likely to admit that they had less knowledge about ASF
	Hunting experience	<0.001 *	Older age groups were more likely to state that they had more hunting experience than younger age groups
	Hunting frequency	0.454	
Knowledge about ASF	Hunting experience	0.581	
	Hunting frequency	0.321	
Respondents opinion whether it is possible to control and eliminate ASF	Hunting frequency	0.784	
Wild boar female hunting	Age	0.063	
	County	0.487	
	Hunting experience	0.036 *	More experienced hunters were more likely to participate in hunting female wild boar

* Statistically significant results.

## References

[1] Guinat C., Wall B., Dixon L., Pfeiffer D.U. (2016). English Pig Farmers’ Knowledge and Behaviour towards African Swine Fever Suspicion and Reporting. PLoS ONE.

[2] Nurmoja I., Mõtus K., Kristian M., Niine T., Schulz K., Depner K., Viltrop A. (2020). Epidemiological analysis of the 2015–2017 African swine fever outbreaks in Estonia. Prev. Vet. Med..

[3] Guinat C., Reis A.L., Netherton C.L., Goatley L., Pfeiffer D.U., Dixon L. (2014). Dynamics of African swine fever virus shedding and excretion in domestic pigs infected by intramuscular inoculation and contact transmission. Vet. Res..

[4] Blome S., Gabriel C., Beer M. (2013). Pathogenesis of African swine fever in domestic pigs and European wild boar. Virus Res..

[5] Sánchez-Vizcaíno J.M., Mur L., Martínez-López B. (2013). African swine fever (ASF): Five years around Europe. Vet. Microbiol..

[6] Oļševskis E., Guberti V., Seržants M., Westergaard J., Gallardo C., Rodze I., Depner K. (2016). African swine fever virus introduction into the EU in 2014: Experience of Latvia. Res. Vet. Sci..

[7] (2010). EFSA Panel on Animal Health and Welfare (AHAW) Scientific Opinion on African Swine Fever. EFSA J..

[8] Costard S., Mur L., Lubroth J., Sanchez-Vizcaino J., Pfeiffer D. (2013). Epidemiology of African swine fever virus. Virus Res..

[9] Taylor R.A., Podgorski T., Simons R.R.L., Ip S., Gale P., Kelly L.A., Snary E.L. (2020). Predicting spread and effective control measures for African swine fever-Should we blame the boars?. Transbound. Emerg. Dis..

[10] Khomenko S., Beltrán-Alcrudo D., Rozstalnyy A., Pinto J., Lubroth J., Martin V., Gogin A., Kolbasov D. (2013). African swine fever in the Russian Federation: Risk factors for Europe and beyond. Empres Watch.

[11] (2014). Evaluation of possible mitigation measures to prevent introduction and spread of African swine fever virus through wild boar. EFSA J..

[12] Miteva A., Papanikolaou A., Gogin A., Boklund A., Bøtner A., Linden A., Viltrop A., Schmidt C.G., Ivanciu C., Desmecht D. (2020). Epidemiological analyses of African swine fever in the European Union (November 2018 to October 2019). EFSA J..

[13] Gavier-Widén D., Ståhl K., Neimanis A.S., Segerstad C.H.A., Gortázar C., Rossi S., Kuiken T. (2015). African swine fever in wild boar in Europe: A notable challenge. Vet. Rec..

[14] Gogin A., Gerasimov V., Malogolovkin A., Kolbasov D. (2013). African swine fever in the North Caucasus region and the Russian Federation in years 2007–2012. Virus Res..

[15] Nurmoja I., Schulz K., Staubach C., Sauter-Louis C., Depner K., Conraths F.J., Viltrop A. (2017). Development of African swine fever epidemic among wild boar in Estonia - two different areas in the epidemiological focus. Sci. Rep..

[16] Boklund A., Dhollander S., Vasile T.C., Abrahantes J.C., Bøtner A., Gogin A., Villeta L.C.G., Gortázar C., More S.J., Papanikolaou A. (2020). Risk factors for African swine fever incursion in Romanian domestic farms during 2019. Sci. Rep..

[17] More S., Miranda M.A., Bicout D., Bøtner A., Butterworth A., Calistri P., Edwards S., Garin-Bastuji B., Good M., EFSA Panel on Animal Health and Welfare (AHAW) (2018). African swine fever in wild boar. EFSA J..

[18] Schulz K., Oļševskis E., Staubach C., Lamberga K., Seržants M., Cvetkova S., Conraths F.J., Sauter-Louis C. (2019). Epidemiological evaluation of Latvian control measures for African swine fever in wild boar on the basis of surveillance data. Sci. Rep..

[19] Danzetta M.L., Marenzoni M.L., Iannetti S., Tizzani P., Calistri P., Feliziani F. (2020). African Swine Fever: Lessons to Learn From Past Eradication Experiences. A Systematic Review. Front. Vet. Sci..

[20] Boklund A., Cay B., Depner K., Földi Z., Guberti V., Masiulis M., Miteva A., More S., Olsevskis E., Šatrán P. (2018). Epidemiological analyses of African swine fever in the European Union (November 2017 until November 2018). EFSA J..

[21] Calba C., Antoine-Moussiaux N., Charrier F., Hendrikx P., Saegerman C., Peyre M., Goutard F.L. (2015). Applying participatory approaches in the evaluation of surveillance systems: A pilot study on African swine fever surveillance in Corsica. Prev. Vet. Med..

[22] Joshi A., Kale S., Chandel S., Pal D.K. (2015). Likert Scale: Explored and Explained. Br. J. Appl. Sci. Technol..

[23] Agresti A., Coull B.A. (1998). Approximate is Better than “Exact” for Interval Estimation of Binomial Proportions. Am. Stat..

[24] Allepuz A., De Balogh K., Aguanno R., Heilmann M., Beltran-Alcrudo D. (2017). Review of Participatory Epidemiology Practices in Animal Health (1980–2015) and Future Practice Directions. PLoS ONE.

[25] Schulz K., Calba C., Peyre M., Staubach C., Conraths F.J. (2016). Hunters’ acceptability of the surveillance system and alternative surveillance strategies for classical swine fever in wild boar—A participatory approach. BMC Vet. Res..

[26] Urner N., Mõtus K., Nurmoja I., Schulz J., Sauter-Louis C., Staubach C., Conraths F.J., Schulz K. (2020). Hunters’ Acceptance of Measures against African Swine Fever in Wild Boar in Estonia. Prev. Vet. Med..

[27] Urner N., Seržants M., Užule M., Sauter-Louis C., Staubach C., Lamberga K., Oļševskis E., Conraths F.J., Schulz K. (2021). Hunters’ view on the control of African swine fever in wild boar. A participatory study in Latvia. Prev. Vet. Med..

[28] Vaclavek P. ASF in the Czech. Republic: Management Experience and Lessons Learnt. Proceedings of the FAO: Regional ASF Wild Boar Management Workshop.

[29] Czech’s Republic State Veterinary Administration. https://www.inspection.gc.ca/DAM/DAM-animals-animaux/STAGING/text-texte/dis_africswine_event_asf_final_report_presentation_2-1_1567794333916_eng.pdf.

[30] Calba C., Goutard F.L., Hoinville L., Hendrikx P., Lindberg A., Saegerman C., Peyre M. (2015). Surveillance systems evaluation: A systematic review of the existing approaches. BMC Public Heal..

[31] Jori F., Chenais E., Boinas F., Busauskas P., Dholllander S., Fleischmann L., Olsevskis E., Rijks J., Schulz K., Thulke H. (2020). Application of the World Café method to discuss the efficiency of African swine fever control strategies in European wild boar (Sus scrofa) populations. Prev. Vet. Med..

[32] Vergne T., Guinat C., Petkova P., Gogin A., Kolbasov D., Blome S., Molia S., Ferreira J.P., Wieland B., Nathues H. (2014). Attitudes and Beliefs of Pig Farmers and Wild Boar Hunters Towards Reporting of African Swine Fever in Bulgaria, Germany and the Western Part of the Russian Federation. Transbound. Emerg. Dis..

