# Supplementary material for: What Do Lithuanian Hunters Think of African Swine Fever and Its Control—Perceptions
Source: Animals (Basel). 2021 Feb 18;11(2):525. doi: 10.3390/ani11020525 (PMC7922269; doi:10.3390/ani11020525)
Supplement: Supplementary file 1 [file animals-11-00525-s001.pdf]

FRIEDRICH-LOEFFLER-INSTITUT
FLI
Bundesforschungsinstitut für Tiergesundheit
Federal Research Institute for Animal Health
Institut für Epidemiologie

LITHUANIAN UNIVERSITY OF HEALTH SCIENCES

Baltisch-Deutsches HOCHSCHULKONTOR

Questionnaire-No:
(will be filled by the researchers)

Control of African swine fever (ASF) in wild boar

Hunters and their willingness to support ASF control measures are crucial for effectively combating the disease in wild boar. An active participation of hunters requires their understanding and their acceptance of the implemented control measures.

In the following questionnaire, we would like to identify impediments, concerns and opinions regarding the support of ASF control measures. Thus, we would like to ask you as hunters for your thoughts and opinions to increase the motivation needed for ASF control in wild boar.

By filling in this questionnaire you agree that the obtained data is used anonymously for research and publication.

Thank you very much for your participation!

Part I. General information

**Date:**				
**Gender:**	☐ Female	☐ Male	☐ Diverse	
**Age-group (years):**	☐ 18-40	☐ 41-60	☐ > 60	
**County:**	☐ Alytus	☐ Kauno	☐ Klaipėdos	
	☐ Marijampolės	☐ Panevėžio	☐ Šiaulių	
	☐ Tauragės	☐ Telšių	☐ Utenos	
	☐ Vilniaus			
**Hunting frequency (per month):**	☐ less than once	☐ 1-5 times	☐ 6-10 times	☐ more than 10 times
**Hunting experience (years):**	☐ < 5	☐ 5-10	☐ > 10	

Part II. Knowledge about ASF

Question 1:

Please tick the response that best describes the extent to which you agree or disagree with the following statement: My knowledge about ASF is good.

☐ Strongly agree ☐ To a high extent ☐ Neither agree nor disagree ☐ To a low extent ☐ Strongly Disagree

Question 2:

What are your sources of information about ASF (multiple answers possible)?			
☐	State Food and Veterinary Service	☐	Hunting magazine
☐	Private veterinarians	☐	Newspaper and TV
☐	Official hunting meetings	☐	Hunting colleagues
☐	Other, please specify:		

____

Part III. Control of ASF (vaccination is currently not available, thus is it not listed as a control option)

Question 3:

In what kind of actions to control ASF did you take part (multiple answers possible)?			
☐	Intensified hunting	☐	Sampling of wild boar
☐	Compulsory active carcass search	☐	Voluntary active carcass search
☐	Disposal of carcass	☐	Selective hunting on female wild boar
☐	Performing increased biosecurity measures incl. disinfection, installation of disinfection barriers, fencing of dressing places	☐	None (then continue with question **5**)
☐	Other, please specify:		

____

Question 4:

When did you take part in one of the activities listed in question **3**?			
☐	2014/15	☐	2017/18
☐	2015/16	☐	2018/19
☐	2016/17	☐	2019/20

Question 5:

Do you believe, control and elimination of ASF in Lithuania can be achieved?			
☐	Yes	☐	No

Question 6:

Could you imagine that ASF disappears without any human intervention from the Lithuanian wild boar population?			
☐	Yes	☐	No

Question 7:

To which extend do you consider the listed control measures as effective in eliminating ASF from the wild boar population (independently of their feasibility)?

	Very effective	To a high extent	Neither effective nor ineffectiv	To a low extent	Not effective at all
Reduction of population density through intensified hunting	☐	☐	☐	☐	☐
Search for carcasses and removal	☐	☐	☐	☐	☐
Selective hunting on females	☐	☐	☐	☐	☐
Ban of individual hunting	☐	☐	☐	☐	☐
Ban of driven hunting					
Including additional forces like army/police/hired hunters in active carcass finding	☐	☐	☐	☐	☐
Other additional forces:					
Including additional forces as described above in wild boar shooting	☐	☐	☐	☐	☐
Other additional forces:					
Increased biosecurity measures	☐	☐	☐	☐	☐
Ban of supplementary feeding	☐	☐	☐	☐	☐
Provision of supporting hunting tools like night vision or silencer					
Other, please specify: ____________________	☐	☐	☐	☐	☐
Other, please specify: ____________________	☐	☐	☐	☐	☐

Question 8:

Which options do you consider as **not** feasible in the field and why (multiple answers possible)?

☐ Including additional forces like army or police; why: ____________________
☐ Selective hunting on females; why: ____________________
☐ Ban of hunting; why: ____________________
☐ Increased biosecurity measures; why: ____________________
☐ Ban of supplementary feeding; why: ____________________
☐ Other; why: ____________________
Please specify: ____________________
____
☐ None of the above, because all are feasible

Question 9:

What are the hindering factors in supporting the control and elimination of ASF in wild boar (multiple answers possible)?

☐ Time constraints
☐ Do not know how I could support it
☐ Lack of trust in the authorities
☐ I do not consider ASF as a problem
☐ Additional costs
☐ I do not believe this is possible

Part IV. Enhanced passive surveillance (active search and reporting of carcasses)

Question 10:

	Very	To a high extent	Neither nor	To a low extent	Not at all
**Looking for carcasses, at times when you are out in the forest anyway (e.g. for hunting):**					
How do you assess the **effectiveness**?	☐	☐	☐	☐	☐
How **willing** are you to support this?	☐	☐	☐	☐	☐
**Going actively out into the forest to focus specifically on the carcass search:**					
How do you assess the **effectiveness**?	☐	☐	☐	☐	☐
How **willing** are you to support this **if you get paid?**	☐	☐	☐	☐	☐
How **willing** are you to support this **without any financial compensation?**	☐	☐	☐	☐	☐

Question 11:

What are the hindering reasons to support carcass search and reporting (multiple answers possible)?

- ☐ Time constraints
- ☐ Lack of personal benefit
- ☐ Additional costs
- ☐ Requirement to bury the carcasses by hunters
- ☐ Difficulties to find dead wild boar
- ☐ I do not believe that carcass finding will help to eliminate ASF
- ☐ I do not believe control and elimination is possible
- ☐ Other, please specify: ______________________

Question 12:

What options would increase your motivation to support passive surveillance (multiple answers possible)?

- ☐ Personal financial support
- ☐ Personal support from additional forces (reduction of work)
- ☐ Availability of trained dogs for carcass finding
- ☐ Detailed feedback from the authorities regarding the disease status of the wild boar
- ☐ No further work than reporting the location of the dead wild boar
- ☐ Other, please specify: ______________________

Comments:

Is there anything else you would like to tell us?

____

____

____

Thank you for your participation! Your time is greatly appreciated and has contributed to our research.
